# Surface-Treated MDI-Compatibilized PPC-P/PPC-ECH Film with PVA/Tannic Acid Complex for High-Gas-Barrier Application

**DOI:** 10.3390/polym18040520

**Published:** 2026-02-20

**Authors:** Shuangshuang Yue, Jiangtao Deng, Guoshan He, Wanjuan Wang, Min Xiao, Sheng Huang, Shuanjin Wang, Dongmei Han, Yuezhong Meng

**Affiliations:** 1 School of Chemical Engineering and Technology, Sun Yat-sen University, Guangzhou 510275, China; 2 Key Laboratory of Low-Carbon Chemistry & Energy Conservation of Guangdong Province, State Key Laboratory of Optoelectronic Materials and Technologies, School of Materials Science and Engineering, Sun Yat-sen University, Guangzhou 510275, China; 3 Key Laboratory of Guangdong Higher Education Institutions of Northeast Guangdong New Functional Materials, School of Chemistry and Environment, Jiaying University, Meizhou 514015, China; 4 Guangzhou Quality Testing and Inspection Institute, Collaborative Innovation Center for NQI-Quality Safety of Guangzhou, Technology Innovation Center of Food Related Product Quality and Safety, State Administration for Market Regulation, Guangzhou 510000, China; 5 Institute of Chemistry, Henan Academy of Sciences, Zhengzhou 450000, China

**Keywords:** biodegradable, composites, coating materials, barrier property

## Abstract

A novel low-cost poly(propylene carbonate-co-epichlorohydrin) (PPC-ECH) with mechanical properties similar to those of poly (butylene adipate-co-terephthalate) (PBAT) was developed and incorporated into a poly(propylene carbonate-co-phthalate) (PPC-P) matrix. Meanwhile, 4, 4′-diphenylmethane diisocyanate (MDI) was employed as a reactive compatibilizer and mixed with PPC-P and PPC-ECH to create a variety of PPC-P/PPC-ECH/MDI blends. The effects of PPC-ECH and MDI content on the mechanical, optical, thermal, morphological, and gas barrier properties of the blends were systematically investigated. Results demonstrated that MDI reacts with both PPC-P and PPC-ECH, forming a chemically bonded interface that significantly improves their compatibility. Notably, when 2 phr of MDI was incorporated, the elongation at break of the PPC-P/PPC-ECH/2MDI blend increased dramatically from 71% to 502%, while maintaining good tensile strength (~23 MPa) and light transmittance (~80%). To further enhance the gas barrier performance, a high-oxygen-barrier poly(vinyl alcohol) (PVA)/tannic acid (TA) complex coating was applied to the surface of the PPC-P/PPC-ECH/2MDI film. This coating synergistically leveraged the abundant hydroxyl groups in PVA and TA to form a dense hydrogen-bonded network, reducing oxygen permeability to an ultra-low value of 0.1 cm^3^·mm/(m^2^·day). This outstanding performance highlights the strong potential of PPC-P/PPC-ECH-based films for advanced packaging applications.

## 1. Introduction

Poly(propylene carbonate-co-phthalate) (PPC-P) is a kind of biodegradable semi-aromatic poly(ester-co-carbonate) synthesized by alternating copolymerization of phthalic anhydride (PA), carbon dioxide (CO_2_), and propylene oxide (PO) [1]. Advantages such as biodegradability, biocompatibility, transparency, and gas barrier property impart PPC-P with great application potential in fields of packaging, tableware, foaming, and plastic film [2,3,4,5]. However, the current PPC-P application is still limited by its inherent brittleness and low impact resistance, making it challenging to use in circumstances where considerable toughness is required. To address the inherent brittleness of PPC-P while preserving its excellent biodegradability, a chain extender (triphenylmethane tri-isocyanate, TTI) was introduced to chemically modify the polymer, which brought marginal improvements in mechanical performance, with an increase in tensile strength from 46.8 MPa to 49.3 MPa and the elongation at break from 4.9% to 13.5% [3]. Another effective way is to modify PPC-P with inherently flexible biodegradable plastics, such as poly(butylene adipate-co-terephthalate) (PBAT) [6]. Zhang et al. prepared a series of PPC-P/PBAT blends via melting blending and found that a mass ratio of 70/30 (PPC-P/PBAT) provided an optimal balance between strength and toughness. However, due to the poor compatibility between PPC-P and PBAT, the elongation at break of PPC-P/PBAT blends (70/30 (*w*/*w*)) only increased to 34.8%, which remains insufficient for practical applications [7]. One efficient way to boost the performance of blend materials is to add small molecules with interaction groups as a reactive compatibilizer [8,9,10,11]. This approach improves interfacial compatibility between blend components, thereby optimizing the material’s overall performance. Song et al. [12] used epoxidized soybean oil (ESO) to compatibilize PLA/PPC blends, and found that an appropriate amount of ESO (5 phr) improved the compatibility of PLA/PPC blends (70/30) through ring-opening reaction and chain extension, thereby enhancing their mechanical properties. The compatibilized blend exhibited an elongation at break 4.8 times greater than that of the unmodified PLA/PPC blend. In our previous work [6], we developed glycerol@4,4′-diphenylmethane diisocyanate (MDI@Gly) as a compatibilizer for PPC-P/PBAT blends and found that MDI@Gly reacts efficiently with the terminal OH groups of both PPC-P and PBAT, thereby significantly improving the interfacial affinity between PPC-P and PBAT. The compatibilized PPC-P/PBAT blend (75/25, wt/wt) exhibited remarkable mechanical enhancement when the content of MDI@Gly is 4 wt%, achieving an elongation at break of 435%, which is 45.3 times that of the unmodified blend. It expands the application prospects of PPC-P/PBAT blends in packaging. However, the market price of PBAT is relatively high, which will increase the application cost of PPC-P/PBAT blends.

Recently developed PPC-ECH (structure shown in Figure S3), a novel material synthesized through ternary copolymerization of PO, ECH, and CO_2_ [13], exhibits mechanical properties comparable to PBAT while offering additional advantages, including lower cost, superior gas barrier property, and a molecular structure analogous to PPC-P. These characteristics suggest that blending PPC-P with PPC-ECH could yield a polymer composite with outstanding overall performance, making it particularly suitable for packaging applications.

In this study, we strategically replaced PBAT with PPC-ECH as the blending component for PPC-P, leveraging their shared poly(propylene carbonate) backbone for enhanced intrinsic miscibility. Crucially, we opted to use pure MDI directly as a compatibilizer, rather than the MDI@Gly [6] employed in previous PBAT/PPC-P systems. This deliberate choice was based on two key considerations: (1) the structural similarity of PPC-P and PPC-ECH reduces the need for a flexible “molecular bridge,” eliminating the benefit of glycerol’s chain-softening effect; and (2) pure MDI enables a more direct and efficient reaction with the terminal hydroxyl groups of both PPC variants, avoiding the unnecessary consumption of -NCO groups and the introduction of overly soft segments that could compromise mechanical properties. By changing the addition amount of PPC-ECH and MDI in the PPC-P matrix, a series of PPC-P/PPC-ECH/MDI blends were prepared. The effects of different PPC-ECH and MDI addition amounts on the thermal, optical, mechanical, and gas barrier properties of the PPC-P/PPC-ECH/MDI blends were studied.

To further enhance the gas barrier properties of the blends, we constructed a high-oxygen-barrier poly(vinyl alcohol) (PVA)/tannic acid (TA) complex coating [14] on the surface of the polymer blends. They are chosen for several key reasons: (1) TA, a natural polyphenol and food-grade substance, is widely available in nature and readily biodegradable by soil bacteria [15,16]. (2) PVA has outstanding biocompatibility, transparency, and oxygen barrier properties [17,18,19,20]. Moreover, both PVA and TA possess abundant −OH groups, enabling strong hydrogen-bonding interactions within the PVA/TA complex coating that are expected to effectively suppress oxygen permeability.

## 2. Experimental Section

### 2.1. Fabrication of PPC-P/PPC-ECH Blend

**Figure 1:**
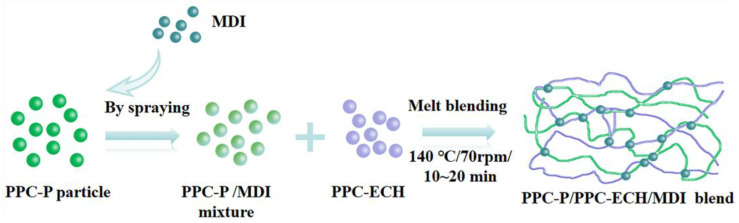
Schematic diagram for the fabrication of PPC-P/PPC-ECH/MDI blends.

PPC-P and PPC-ECH particles were dried under vacuum at 80 °C overnight before use. The initial material ratios used and the process for preparing the blend films are shown in Table 1 and Figure 1. All samples were prepared by reactive blending in a melt mixer (RM-200) with a screw speed of 70 rpm at 140 °C for 10~20 min. Neat PPC-P and PPC-ECH were subjected to the same mixing treatment to have the same thermal history as the blends. All samples were hot-pressed into thin sheets under 10 MPa and 160 °C for 5 min for tests.

### 2.2. Fabrication of PVA/TA Coated Blend Films

**Figure 2:**
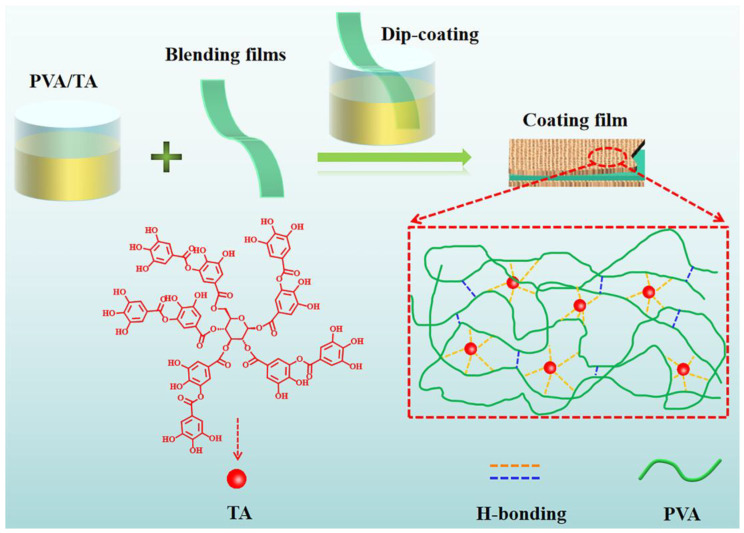
Schematic illustration of the construction process of PVA/TA complex coating on the surface of the blend film.

PVA was dissolved in 90 °C deionized water and cooled to room temperature to form a transparent solution with a concentration of 1~6.0 wt%. Under vigorous stirring, the TA was added to the PVA solution to prepare the PVA/TA complex solutions with different formulations. The initial material ratios used to prepare the coating solution are shown in Table 2. The coating process is illustrated in Figure 2. The blend film (60PPC-P/PPC-ECH) was dipped into the coating solutions for 2 min, withdrawn vertically, and then dried at 40 °C for 1 h. The coated layers were denoted as PVA*x* and PVA*x*/TA*y*, where x and y represent the concentration of PVA and TA, respectively.

## 3. Results and Discussion

### 3.1. Optimization of Blending Temperature

**Figure 3:**
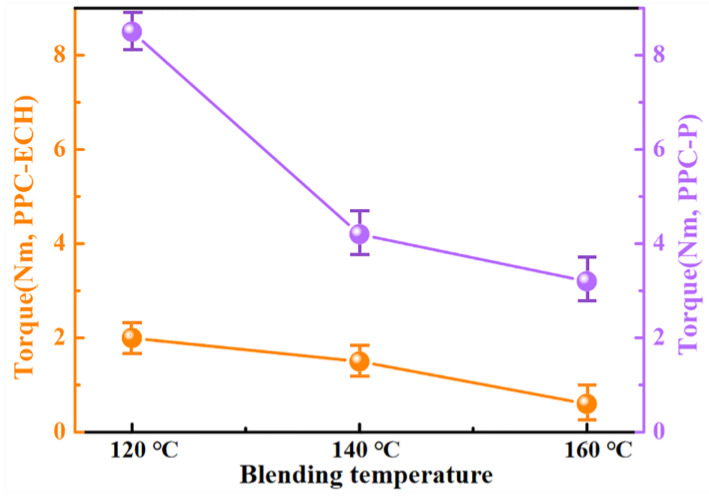
Torque values of polymers at various temperatures.

Temperature plays a critical role in determining the viscosity differential between the two polymer components during blending, directly impacting the blend’s microscopic phase morphology and interfacial characteristics—factors that ultimately govern the material’s mechanical performance. The viscosity of the polymer at different temperatures is reflected in the real-time torque measurements during the blending process. The torque values of PPC-P and PPC-ECH at various temperatures are displayed in Figure 3. It is apparent that the torque values of PPC-P and PPC-ECH differ greatly at lower temperatures. As the temperature increases to 140 °C, this difference diminishes markedly, suggesting improved viscosity matching. However, further increasing the temperature to 160 °C does not lead to additional convergence in torque values. These results indicate that the melt viscosities of PPC-P and PPC-ECH are most closely matched within the 140–160 °C range, promoting optimal mixing compatibility. Therefore, 140 °C was chosen as the optimal blending temperature for PPC-P and PPC-ECH.

### 3.2. Mechanical Properties of PPC-P/PPC-ECH Blends

**Figure 4:**
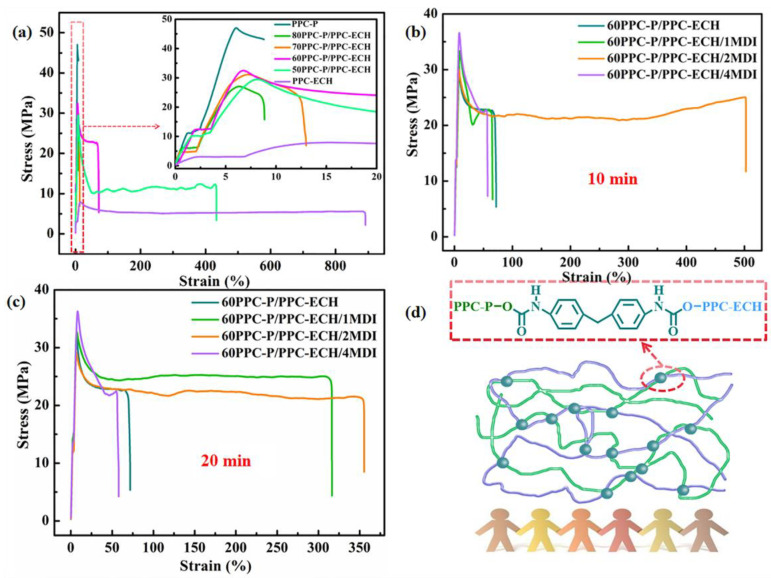
(**a**) Effect of PPC-ECH proportions on tensile property of PPC-P/PPC-ECH binary blends. (**b**,**c**) Effect of MDI on tensile properties of PPC-P/PPC-ECH binary blends with different blending times. (**d**) Proposed schematic diagram of PPC-P/PPC-ECH blends after incorporating MDI.

Figure 4a shows the tensile curves of the PPC-P/PPC-ECH blends with various weight ratios. The neat PPC-P, which was designed as the main matrix, possessed a tensile strength of 47.4 MPa and an elongation at break of 7.3%, respectively. The PPC-ECH was added as a modifier (≤50 wt%) to optimize the mechanical properties of PPC-P. With the increase in PPC-ECH content, the elongation at break of PPC-P/PPC-ECH blends decreased continuously, while the tensile strength reduced sharply. When the content of PPC-ECH increased to 40%, the 60PPC-P/PPC-ECH exhibited an elongation at break of 71%, which was 10 times that of the neat PPC-P, while the break strength remained 22.5 MPa. With the addition of PPC-ECH, further increasing to 50%, the elongation at break of the blend reached 433%. Unfortunately, the break strength decreased sharply to 12.2 MPa. Since the 60PPC-P/PPC-ECH blend exhibited acceptable elongation at break and tensile strength, MDI was incorporated into the system to enhance interfacial compatibility and further improve mechanical performance.

When MDI was incorporated into the 60PPC-P/PPC-ECH blends, the elongation at break was improved greatly while maintaining a high tensile strength, leading to a strikingly enhanced toughness (Figure 4b,c). As shown in Figure 4b, increasing the MDI content from 0 to 2 phr caused the elongation at break of 60PPC-P/PPC-ECH/2MDI to surge from 71% to 502%, with no compromise in tensile strength. These findings indicate that MDI’s compatibilizing effect strengthened molecular interactions within the blends and facilitated network structure formation (Figure 4d). However, further increasing MDI content to 4 phr led to reduced elongation at break. This phenomenon can be attributed to the formation of a highly crosslinked network structure, where the improved dispersion of MDI at higher dosages leads to a crosslinking density so elevated that it significantly restricts molecular chain mobility, thereby diminishing the material’s elongation capability. Furthermore, achieving optimal performance requires careful balancing of MDI dosage and mixing time. When the blending time was extended to 20 min, the elongation at break reached 316% even at a low MDI content of 1 phr. However, when 2 phr of MDI was added, the elongation at break decreased compared to samples blended for only 10 min. This reduction is likely due to the improved dispersion of MDI achieved with prolonged mixing, which promoted a more extensive and uniform crosslinking reaction. While this enhanced the network’s integrity, it also significantly increased the crosslinking density. The resulting high degree of chain entanglement and restricted chain mobility ultimately led to the observed decline in ductility. Therefore, the 10 min blending time appears to be optimal for the 2 phr MDI system, as it achieves a crosslinking density that maximizes toughness without overly constraining chain movement.

### 3.3. Thermal Properties of PPC-P/PPC-ECH Blends

**Figure 5:**
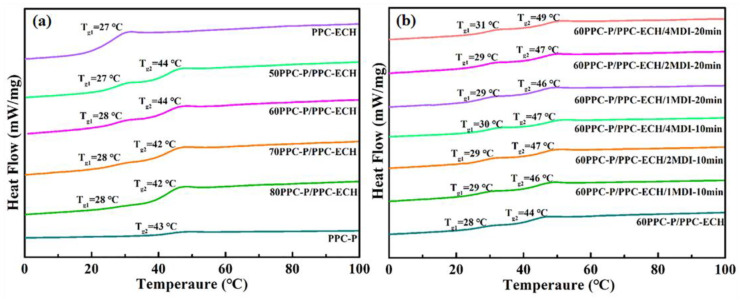
(**a**) DSC curves of neat PPC-P, neat PPC-ECH, and PPC-P/PPC-ECH composites; (**b**) DSC curves of 60PPC-P/PPC-ECH with different content of MDI and blending time.

DSC measurement was conducted to reveal the effect of MDI on the glass transition temperature (*T_g_*) of PPC-P/PPC-ECH blends. Figure 5a depicts the second heating curves of pure PPC-P, pure PPC-ECH, and PPC-P/PPC-ECH blends. The *T_g_* of pure PPC-P and PPC-ECH was 43 °C and 27 °C, respectively, while all PPC-P/PPC-ECH blend samples had two *T_g_*(*T_g1_* and *T_g2_*), corresponding to the *T_g_* of the two phases of PPC-ECH and PPC-P, respectively. After introducing MDI into the 60PPC-P/PPC-ECH blend, as the amount of MDI in the system increases, the *T_g_* of the PPC-P and the PPC-ECH phase all gradually increase. This indicates that MDI acts as a chain extender, helps extend PPC-P chains, PPC-ECH chains, as well as linking some PPC-ECH flexible chains to PPC-P molecular chains. Due to the strengthening of chain entanglement caused by chain expansion, the movement of chain segments is restricted, resulting in an increase in the *T_g_* of both phases. In addition, the MDI bridged PPC-P-*b*-PPC-ECH chains can act as a compatibilizer, improving the compatibility of 60PPC-P/PPC-ECH blends, therefore increasing the mechanical properties [21,22,23,24].

**Figure 6:**
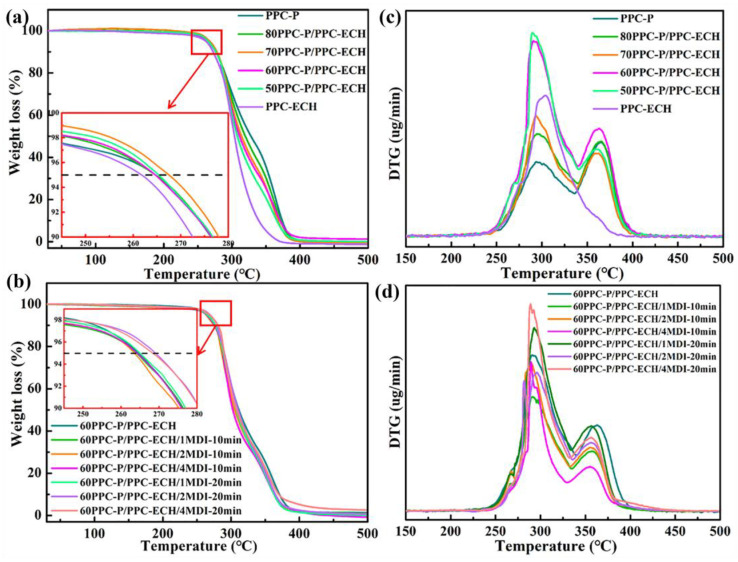
Thermogravimetric analysis curves of PPC-P, PPC-ECH, PPC-P/PPC-ECH, and 60PPC-P/PPC-ECH/MDI blends with different amounts of MDI added (**a**,**b**) TG curves; (**c**,**d**) DTG curves.

Figure 6 shows the thermogravimetric analysis curves of PPC-P, PPC-ECH, PPC-P/PPC-ECH and 60PPC-P/PPC-ECH/MDI blends. Figure 6a,c is the TGA curve, and Figure 6b,d is the DTG curve, with related data shown in Table 3. As can be seen from Figure 6a and Table 3, the T_d5%_/T_d95%_ of pure PPC-P and PPC-ECH were 261 °C/380 °C and 258 °C/351 °C, respectively. The T_d5%_/T_d95%_ of PPC-P/PPC-ECH is distributed between that of PPC-P and PPC-ECH. However, increasing the PPC-ECH content has no meaningful effect on the blend’s T_d5%_. Furthermore, PPC-P has two maximum thermal breakdown temperatures (T_dmax1_ and T_dmax2_), which correspond to the polycarbonate and polyester chain segments, respectively. As a result, the T_d95%_ blends are more similar to PPC-P because thermal disintegration of polyester segments is the main process at late stages.

From Figure 6b,d, it can be seen that the decomposition curve of the 60PPC-P/PPC-ECH blend after adding MDI is roughly similar to the decomposition curve of the blend without MDI, both with two steps, corresponding to the thermal degradation of the polycarbonate and polyester segments, respectively. As can be seen from Table 3, there was no significant change in T_d5%,_ and T_dmax_ when MDI is introduced into the 60PPC-P/PPC-ECH blend. All the samples exhibit a T_d5%_ higher than 263 °C, which is far above the processing temperature of the PPC-P/PPC-ECH blend (140 °C).

### 3.4. Optical Properties Analysis

**Figure 7:**
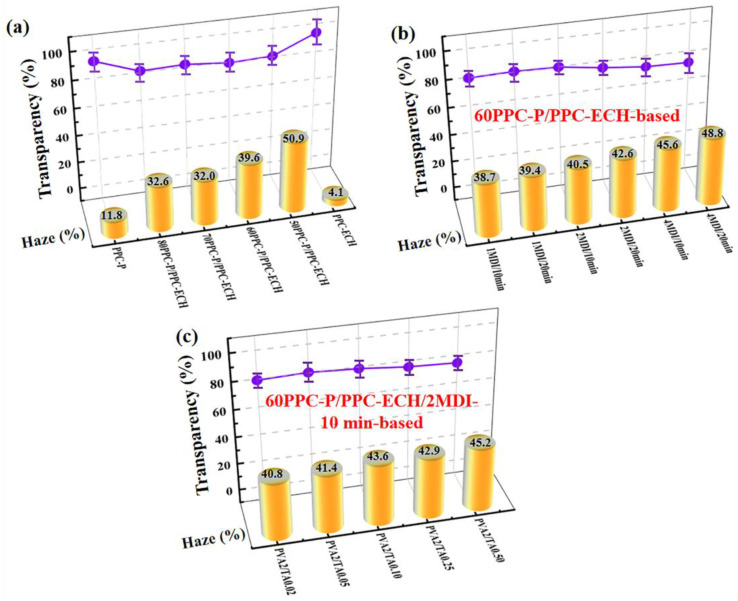
(**a**) Haze and light transmittance of pure PPC-P, PPC-ECH, and PPC-P/PPC-ECH blends; (**b**) haze and light transmittance of 60PPC-P/PPC-ECH/MDI blends; (**c**) haze and light transmittance of the PVA/TA complexes coated 60PPC-P/PPC-ECH/2MDI-10 min films.

Haze and visible light transmission are two crucial indicators for evaluating the optical performance of packaging material [25,26,27]. As shown in Figure 7a, the pristine PPC-P and PPC-ECH films exhibited excellent optical clarity, demonstrating light transmittance values of 91.7% and 93.5%, with corresponding haze measurements of 11.8% and 4.1%, respectively. However, the optical properties of PPC-P/PPC-ECH blends demonstrated significant phase-dependent behavior. The light transmittance of all blended samples was markedly lower than that of pure PPC-P and PPC-ECH, while the haze values showed a progressive increase with higher PPC-ECH content.

The influence of MDI content on the optical characteristics of 60PPC-P/PPC-ECH/MDI blend films is illustrated in Figure 7b. As the MDI content increases, the optical transmittance of the blended system slightly decreases, while the haze increases. This phenomenon primarily stems from MDI’s dual role in the polymer system. While MDI effectively enhances material toughness through strengthened intermolecular interactions, it simultaneously introduces structural heterogeneities, such as refractive index variations between crosslinked and non-crosslinked regions, micro-scale phase separation boundaries, and localized defects in the polymer matrix. These structural modifications collectively enhance light scattering, resulting in the observed optical property changes. Remarkably, both the 60PPC-P/PPC-ECH/2MDI-10 min film and the PVA/TA complex coated 60PPC-P/PPC-ECH/2MDI-10 min film maintain excellent optical performance (Figure 7c), with the PVA_2_/TA_0.10_-coated composite film achieving 80% light transmittance while exhibiting a haze value of 43.6%. This indicates that the coating process effectively maintains optical clarity while potentially providing additional surface functionality.

### 3.5. Coating Layer Properties

**Figure 8:**
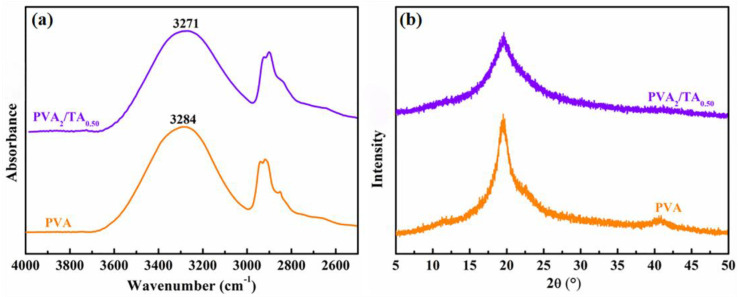
(**a**) ATR-FTIR, (**b**) XRD spectra of pure PVA, TA, and PVA_2_/TA_0.50_.

To elucidate the enhanced hydrogen bonding between PVA and TA, ATR-FTIR spectroscopy was conducted on both pristine PVA and the PVA_2_/TA_0.50_ coating. As shown in Figure 8a, PVA exhibited a broad and intense absorption band centered at 3284 cm^−1^, attributed to the symmetric O-H stretching vibration. In contrast, the O-H stretching band of the PVA_2_/TA_0.50_ coating was significantly red-shifted to 3271 cm^−1^. According to the literature [28], the formation of intramolecular or intermolecular hydrogen bonds reduces the force constant of the O-H bond, resulting in a redshift of the vibrational frequency; a more pronounced shift indicates stronger hydrogen bonding. Thus, the marked red shift observed in the PVA_2_/TA_0.50_ coating provides direct evidence for the formation of stronger hydrogen bonds between PVA and TA. PVA, a semi-crystalline polymer, showed a characteristic XRD pattern (Figure 8b) with diffraction peaks at 2θ = 19.6°, 22.9°, and 40.8°, corresponding to the (101), (200), and (102) planes of PVA microcrystals, respectively [29]. The PVA_2_/TA_0.50_ coating exhibited a similar XRD pattern, confirming the retention of the PVA crystalline structure. However, the calculated crystallinity of the PVA_2_/TA_0.50_ coating (28.64%) was significantly lower than that of pristine PVA (37.98%). This reduction in crystallinity indicates that the incorporation of amorphous TA disrupts the ordered arrangement of PVA molecular chains.

### 3.6. Gas Barrier Properties Analysis

**Figure 9:**
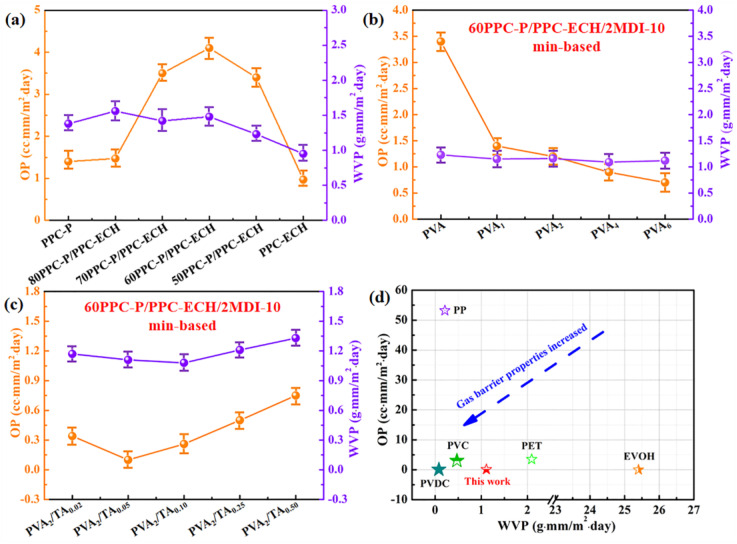
(**a**) OP and WVP values of pure PPC-P, PPC-ECH, and PPC-P/PPC-ECH blends; (**b**) OP and WVP values of PVA solutions of different concentrations coated 60PPC-P/PPC-ECH/2MDI-10 min films; (**c**) OP and WVP values of the PVA/TA complexes coated 60PPC-P/PPC-ECH/2MDI-10 min films; (**d**) Data on the barrier properties of traditional plastic packaging films [33].

Furthermore, the OP and WVP of the films were evaluated with consideration of their vital significance for practical application in the packaging field [30,31,32]. As demonstrated in Figure 9a, PPC-P and PPC-ECH exhibited excellent gas barrier properties, with OP and WVP values of 1.4/0.97 cm^3^·mm/m^2^·day and 1.38/0.95 g·mm/m^2^·day. However, the blending of PPC-P with PPC-ECH resulted in compromised barrier performance. This deterioration stems from the thermodynamic incompatibility between the two polymers, where blending-induced phase separation creates interfacial regions between dispersed and continuous phases containing poorly entangled molecular chains or microporous defects that promote gas penetration.

PVA is a polymer with superior OP due to the large number of hydroxyl groups on the molecular chain. The application of a PVA coating on the blend film surface significantly enhances its oxygen barrier performance. As demonstrated in Figure 9b, coating the film with a 2 wt% PVA solution followed by drying reduces the OP value from 3.4 cm^3^·mm/(m^2^·day) to 1.2 cm^3^·mm/(m^2^·day). When the coating concentration is increased to 6 wt%, the OP value further decreases to 0.7 cm^3^·mm/(m^2^·day). Notably, the PVA coating has minimal impact on WVP, which can be attributed to PVA’s inherent limitations in water vapor barrier performance and the thin nature of the applied coating layer. These promising results in oxygen barrier enhancement without compromising WVP stability motivated our subsequent investigation into further improving the barrier properties through chemical modification approaches.

Based on the above results and analyses, a 2 wt% PVA was selected for cross-linking with tannic acid (TA) to further increase the gas barrier property of the composite film. This improvement is mainly attributed to the aromatic molecular structure of TA, the abundant -OH groups in the macromolecular chains of PVA, and the hydrogen-bonding interactions between PVA and TA. As shown in Figure 9c, coating the composite film with PVA_2_/TA_0.02_ reduced its OP values to 0.34 cm^3^·mm/m^2^·day. When the concentration of TA was further increased to 0.05 g/100 mL, the oxygen transmission coefficient of the composite film decreased to 0.1 cm^3^·mm/m^2^·day. demonstrating a significant enhancement in barrier performance. Furthermore, the OP value of PVA_2_/TA_0.02_ coated blend films is slightly higher than that of PVDC and EVOH films, but significantly lower than that of high-barrier films such as PVC and PET films (as shown in Figure 9d), and laminated films used in potato chip and instant noodle packaging.

## 4. Conclusions

To enhance the toughness of PPC-P, this study employed PPC-ECH as an alternative toughening material to conventional PBAT, with MDI introduced as a compatibilizer to improve interfacial adhesion between the two phases. Through systematic investigation of the effects of PPC-ECH and MDI content on the thermal, mechanical, optical, and gas barrier properties of the blend system, the 60PPC-P/PPC-ECH/2MDI-10 min film was identified as the optimal formulation, demonstrating superior comprehensive performance. Further improving the gas barrier performance of the composite films was realized by applying a PVA/TA coating on the blend surface. The synergistic interaction between PVA and TA—enabled by TA’s aromatic structure and strong intermolecular hydrogen bonding—resulted in an ultra-low oxygen permeability, achieving high-performance oxygen barrier properties comparable to advanced barrier materials. This work presents a simple yet effective strategy for designing fully biodegradable, all-organic coatings with exceptional oxygen barrier performance using environmentally friendly raw materials. Further research is needed to elucidate the underlying molecular mechanisms and optimize the coating’s performance for broader applications.

## Figures and Tables

**Table 1:** The composition of the PPC-P/PPC-ECH/MDI blends.

Samples	PPC-P (wt%)	PPC-ECH (wt%)	MDI (phr)
PPC-P	100	0	0
80PPC-P/PPC-ECH	80	20	0
70PPC-P/PPC-ECH	70	30	0
60PPC-P/PPC-ECH	60	40	0
50PPC-P/PPC-ECH	50	50	0
PPC-ECH	0	100	0
60PPC-P/PPC-ECH/1MDI	60	40	1
60PPC-P/PPC-ECH/2MDI	60	40	2
60PPC-P/PPC-ECH/4MDI	60	40	4

**Table 2:** The content of each component in the coating complex solutions.

Samples	PVA (g)	TA (g)	H_2_O (mL)
PVA	0	0	100
PVA_1_	1	0	100
PVA_2_	2	0	100
PVA_4_	4	0	100
PVA_6_	6	0	100
PVA_2_/TA_0.02_	2	0.02	100
PVA_2_/TA_0.05_	2	0.05	100
PVA_2_/TA_0.10_	2	0.10	100
PVA_2_/TA_0.25_	2	0.25	100
PVA_2_/TA_0.50_	2	0.50	100

**Table 3:** Thermogravimetry data of PPC-P/PPC-ECH 60PPC-P/PPC-ECH/MDI blends.

Sample	T_d5%_	T_dmax1_/T_dmax2_	T_d95%_
PPC-P	261	295/365	380
80PPC-P/PPC-ECH	264	295/365	379
70PPC-P/PPC-ECH	267	294/361	375
60PPC-P/PPC-ECH	265	290/363	381
50PPC-P/PPC-ECH	261	290/361	375
PPC-ECH	258	307/--	351
60PPC-P/PPC-ECH/1MDI-10 min	264	291/357	374
60PPC-P/PPC-ECH/2MDI-10 min	263	290/356	374
60PPC-P/PPC-ECH/4MDI-10 min	264	290/355	375
60PPC-P/PPC-ECH/1MDI-20 min	264	293/357	372
60PPC-P/PPC-ECH/2MDI-20 min	269	296/357	376
60PPC-P/PPC-ECH/4MDI-20 min	268	289/356	403

Note: T_d5%_ is the temperature at which the sample loses 5% of its mass, T_max_ is the temperature at which the sample has the maximum weight loss rate, and T_d95%_ is the temperature at which the sample loses 95% of its mass.

## Data Availability

The raw data supporting the conclusions of this article will be made available by the authors on request.

## Supplementary Materials

The Supporting Information is available free of charge at https://www.mdpi.com/article/10.3390/polym18040520/s1, Figure S1: The synthetic methodology for the tetranuclear organoboron catalysts; Figure S2: ^1^H NMR spectra of synthesized compounds. (a) Tetranuclear boron catalyst intermediate; (b) tetranuclear boron catalyst; Figure S3: Synthetic procedure of PPC-ECH; Figure S4: ^1^H NMR spectrum of PPC-ECH in CDCl_3_; Table S1: Copolymerization of PO/ECH/CO2 with ^Tetra^B.
