# Supplementary material for: Surface-Treated MDI-Compatibilized PPC-P/PPC-ECH Film with PVA/Tannic Acid Complex for High-Gas-Barrier Application
Source: Polymers (Basel). 2026 Feb 20;18(4):520. doi: 10.3390/polym18040520 (PMC12944174; doi:10.3390/polym18040520)
Supplement: Supplementary file 1 [file polymers-18-00520-s001.zip › polymers-4149957-supplementary.pdf]

Supporting Information

Surface-Treated MDI-Compatibilized PPC-P/PPC-ECH Film with PVA/Tannic Acid Complex for High-Gas-Barrier Application

Experimental Section:

Materials

All chemicals were used as received unless otherwise mentioned. PPC-P (Mn=78.6 kg/mol, PDI=1.35) was provided by Shandong Lecron Industrial Development Group, Co., Ltd, China. Propylene oxide (PO) and epichlorohydrin (ECH) were refluxed over CaH_2 for 24 h and distilled prior to use. High purity CO_2 (Guangqi Gas Co. Ltd., > 99.999%), anhydrous acetonitrile (Aladdin,99.8%, H_2O: ≤0.005%), triallyl amine (Energy, 99%), allyl bromide (Aladdin, 98%), 9-borabicyclo[3.3.1]nonane (9-BBN; Energy, 0.5 mol/L in THF) were utilized asreceived.

Characterization

^1H NMR spectra were measured on a Bruker Advanced III 400 MHz NMR spectrometer using $CDCl_3$ or D_2O as solvent. Molecular weight and polydispersity index (PDI) determinations were carried out at 40 °C on a Waters gel permeation chromatography (GPC) system using tetrahydrofuran as eluent and a series of polystyrene (PDI = 1.02) as standards with a flow rate of 1.0 mL/min. The glass transition temperatures (Tg) were determined by differential scanning calorimeter (DSC, Netzsch Model 204). The measurements were carried out at a heating and a cooling rate of 10 °C/min from -100 °C to 180 °C under nitrogen atmosphere. TGA measurements were performed in a Perlin Elmer Pyris Diamond TG/ DTA analyzer under nitrogen atmosphere at a heating rate of 10 °C/min in the temperature range of 30–500 °C. The static mechanical properties of the CO_2-based terpolymers were performed at 25 ◦C and relative humidity of 50 % ± 5 % using a computer-controlled Instron mechanical tester (Model 5566) according to the ASTME-104 standard. The crosshead speed employed was 50 mm/min. Five specimens of each sample were tested, and the average results were reported. The optical performance test was conducted using a light transmission/fog meter (WGT-S, Shanghai Shengguang Instrument & Meter Co., LTD., Shanghai, China) to test the optical performance of the film. This instrument uses tungsten halide lamps, and the wavelength range of the light emitted is between 350~760 nanometers. The oxygen transmission rate and oxygen permeability coefficient of the film samples were measured using an oxygen permeability tester

(Y210, Guangzhou Biaoji Packaging Equipment Co., Ltd., Guangzhou, China). The experimental temperature was 23 °C, and the test standard was GB/T 19789-2005. The water vapor transmission rate of the film samples was ascertained using the water vapor permeability tester (MOCON3/61, Ametek Trading (Shanghai) Co., Ltd., Shanghai, China). The trial temperature was 23 °C, and the test standard was ASTM F-1249.A thickness gauge was used to measure the film's thickness, and each sample had eight uniformly placed spots to determine the average thickness.

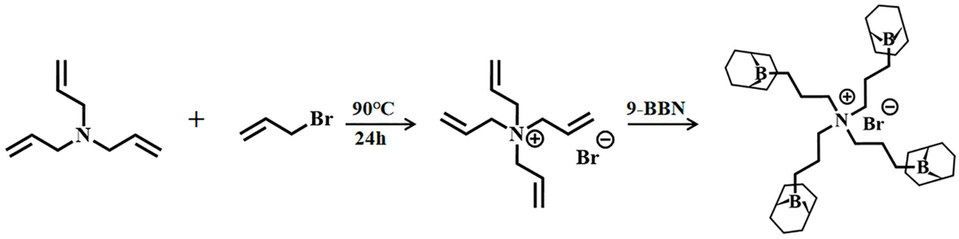

Figure S1. The synthetic methodology for the tetranuclear organoboron catalysts.

Synthesis of tetraallylammonium bromide

In a round-bottomed flask equipped with a stirring bar, 200 mL of anhydrous acetonitrile was added. Subsequently, 13.72 g of triallylamine was dissolved, followed by the dropwise addition of 12.1 g of allyl bromide. The mixture was heated to 90°C and stirred for 24 hours under reflux conditions. After cooling to room temperature, the volatile solvent was removed under reduced pressure. The residue was then washed three times with ethyl acetate to yield the desired white solid quaternary ammonium salt.

Synthesis of organoboron catalysts

To a round-bottomed flask equipped with a stirring bar, add 250 mL of 9-BBN. Subsequently, dissolve 7.1 g of the prepared quaternary ammonium salt. The mixture was heated to 65 °C and stirred for 36 hours under reflux conditions. After cooling to room temperature, the volatile solvent was removed under reduced pressure. The residue was then washed three times with anhydrous n-hexane to afford the desired white solid tetranuclear boron catalyst.

Representative ^{1}H NMR spectra of crude tetraallylammonium bromide and tetranuclear boron catalyst

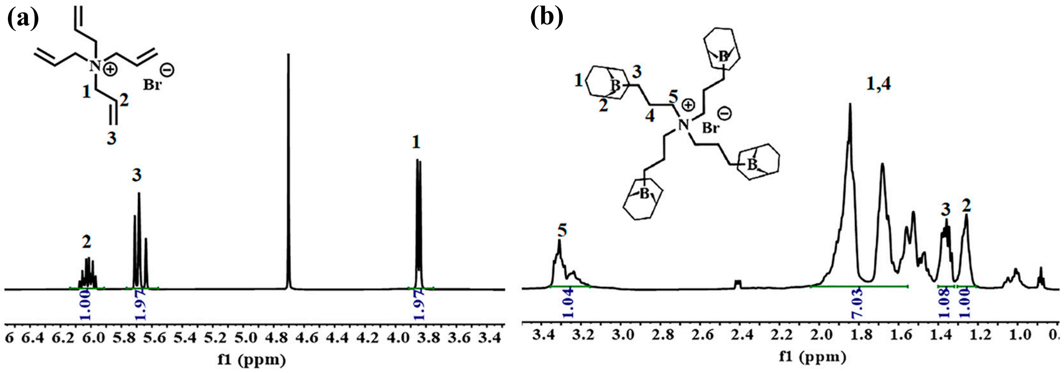

Figure S2. ^{1}H NMR spectra of synthesized compounds.(a)Tetranuclear boron catalyst intermediate; (b)tetranuclear boron catalyst.

General experimental procedures for the copolymerization of CO_2, PO and ECH

A 200 mL Parr reactor with a mechanical stirrer and a small Teflon vial inside was dried in an oven at 110 °C overnight and then immediately placed into the glove box chamber. After pumping for at least 4 h, the reactor was taken into the glove box under N_2 atmosphere. The molar feed during the reaction is PO:ECH:Cat.=1000:200:1. The reactor was sealed and taken out of the glove box. The copolymerization was kept in 45 °C for 20 h at a constant 2.5 MPa CO_2 pressure. At the end of the copolymerization, the reactor was cooled in an ice water bath and the unreacted CO_2 was carefully released. A sample of the crude product was taken and prepared for ^{1}H NMR analysis to determine the conversion and selectivity. 2 mL solution of HCl in ethanol (1 M) was added to quench the reaction. The crude product was dissolved in CH_2Cl_2 and precipitated in ethanol. The product was washed with ethanol and dried in vacuum oven at 80 °C to a constant weight.

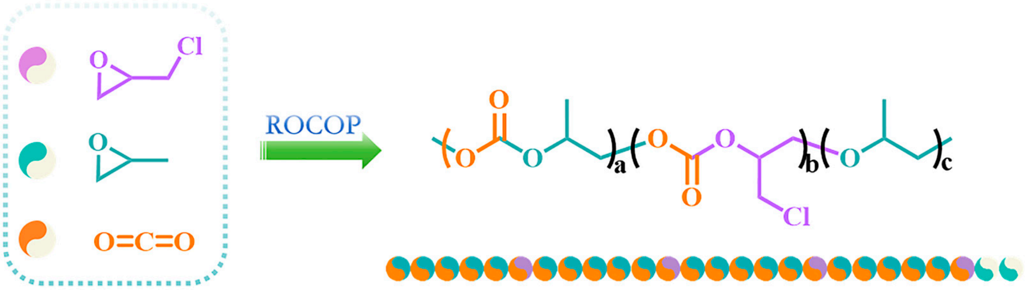

Figure S3. Synthetic procedure of PPC-ECH.

Representative ^{1}H NMR spectra of crude polymerization system.

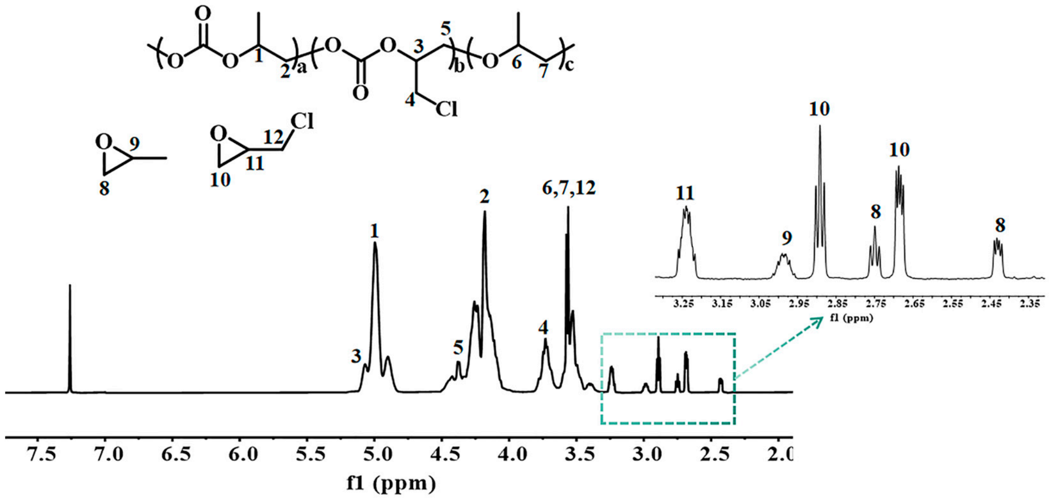

Figure S4. ^{1}H NMR spectrum of PPC-ECH in $CDCl_3$.

ECH/PO/CO_2 Copolymerization Results

Table S1. Copolymerization of PO/ECH/CO_2 with $^{Tetra}B$.[a]

Entry	Sample	PPC/PECHC/PPO (mol%)[b]	PO/ECH (Conv.%)[b]	Mn(kg/mol)/PDI[c]
1	PPC-ECH	68/15/17	80/71	39.0/1.35

[a] PO:ECH:$^{Tetra}B$=1000:200:1，2.5 MPa，40 °C，t=20 h；[b]Determined by ^{1}H NMR spectrum. [c]Determined by GPC.
